# Prevention of Disease. Self-Abnegation

**Published:** 1878-02

**Authors:** Henry S. Chase

**Affiliations:** St. Louis


### PREVENTION OF DISEASE. SELF-ABNEGATION.

BY HENRY S. CHASE, ST. LOUIS.

The writings published and unpublished, of the nobler minds in the dental profession, for the last thirty years, have shown great solicitude in the prevention of dental disease. Besides those who have written, there are thouands of practitioners, who, in the daily practice of their profession give familiar talks on the preservation of the teeth, illustrated by hygienic facts. No body of men have ever so unselfishly attempted to undermine the profession which is giving them their daily bread as the dental profession.

The dental journals have been generously provided with essays on dental hygiene, written in plain language, that it might be appropriated by the simplest minds. The dentist, to-day, considers it his duty to instruct his patients in such a manner, that if the advice is carried out, there would not be in a very short time one dentist needed, where there are ten now. Many dentists have actually seen their income diminish year by year, from the very effect of this hygienic advice.

The public have had dental tracts on hygiene distributed gratuitously. Weekly newspapers of large circulation have contained familiar talks about the teeth, having in view prevention of dental decay. Thousands of dentists purchase dental tracts in large numbers, whose principles have been endorsed by some state dental society, and distribute them among their patients.

What has been the result? A vast improvement in the growth and preservation of the natural teeth.

Take for instance, the advocacy of a proper diet for the nourishment of growing dentos.

Thousands upon thousands now use breadstuffs not demineralized by the miller. Where families formerly used fine flour, they now use unbolted wheat meal, corn meal, oat meal cracked wheat and rye meal.

Twenty-five years ago you might ask in vain for these foods at hotels and restaurants; now they are all on the "bill of fare." The good work of instruction still goes on, for there are millions who do not yet come within reach of dental advice, but are coming within its influence gradually.

What insures the health of the teeth, insures that of the general health, also, and I therefore claim that the dental profession has done more for reform in diet than all other influences put together. For the truth of this I refer to dental literature for the last thirty years.

A few months ago, I said twenty years from now no more dentists will be needed in the United States than we have to-day. It is true then, that the dental profession is to-day, and has been for twenty years past, advocating measures, which, if carried out, will destroy the foundation on which it is built.

A noble mind will not regret this, but rather rejoice that humanity may be freed from one of the greatest ills of life, namely, dental decay.

